# Editorial: Wildlife Welfare

**DOI:** 10.3389/fvets.2020.576095

**Published:** 2020-09-30

**Authors:** Charlotte Berg, Henrik Lerner, Andrew Butterworth, Chris Walzer

**Affiliations:** ^1^Department of Animal Environment and Health, Swedish University of Agricultural Sciences, and the Swedish Centre for Animal Welfare (SCAW), Skara, Sweden; ^2^Department of Health Care Sciences, Ersta Sköndal Bräcke University College, Stockholm, Sweden; ^3^Independent Veterinary Consultant, Welfare Max, Bristol, United Kingdom; ^4^Wildlife Conservation Society, Bronx, NY, United States; ^5^Research Institute of Wildlife Ecology, University of Veterinary Medicine Vienna, Vienna, Austria

**Keywords:** wildlife, conservation, animal welfare, captive breeding, 3R, wildlife management

Animal welfare relates to the feelings, behavior, and the health status of animals. Nevertheless, animal welfare legislation rarely prescribes what animals should feel or experience, but rather what humans should do to protect the animals in their care from unnecessary suffering, and e.g., specifications to provide them with suitable housing conditions and appropriate feed to ensure a reasonably good life. This obviously applies to domesticated animals and wildlife kept in enclosures, but not to free-roaming wildlife. Wildlife welfare has received far less attention than welfare for farm or companion animals, although attempts have been made (1, 2). In recent years the extent of interest in wildlife welfare has grown, as more people have realized that humans have a substantial influence on the lives and welfare of wildlife individuals. Humans, as individuals and as a species, intentionally or unintentionally influence the welfare of wildlife in many different ways, some of which are discussed in this special issue.

The growing global human population is impacting wildlife habitats, and causing disturbance or destruction of nature, be it for infrastructure projects such as roads, city expansion or beach resorts, or to gain access to natural resources such as oil, timber or minerals. The expanding human population requires more food. Livestock and feed production are among the greatest threats to biodiversity and key drivers in land-use change. Forests and savannahs are being converted into agricultural land for crop and animal production while oceans are unsustainably trawled for fish. This will inevitably decrease the potential for wildlife to find suitable areas for breeding, foraging, staging during migration or hiding from predators. In this volume, Stephen and Wade present lamprey on Vancouver Island as an aquatic example of how to work with shared priorities for social expectations, conservation obligations and species recovery at the population level of welfare.

By introducing domestic livestock to an area, humans will not only compete for space, but may also contribute to the spread of various infectious diseases from livestock to wild species (and, of course, also the other way around) or from wildlife to humans (3). There is also the obvious threat, not only to the survival of certain species but also to the welfare of individual animals, caused when humans, intentionally or by accident, introduce invasive species to a new region, resulting in predation or inter-species competition for resources such as nesting sites or food. If humans then decide to eradicate such invasive species, the eradication process may in turn involve negative effects on the welfare of the individuals of the invasive species.

Unregulated hunting, poaching and unsustainable fishing by humans can, over time, reduce the number of wild animal individuals to a level where they can no longer proliferate and will become extinct. Such activities can also directly lead to animals being hit or caught, struck and lost, injured but not killed—causing considerable suffering if the animal cannot immediately be located and humanely killed. Furthermore, hunting and fishing activities may impact animals other than the intended prey, through disturbance, by-catch or entanglement. The entanglement of cetaceans is addressed by Dolman and Brakes, where the authors discuss the animal welfare consequences of incidental capture of marine wildlife in commercial fishing gear.

It should be acknowledged that tourism activities, even when carried out by wildlife enthusiasts, who may aim to support wildlife in the wild, may have unintended negative side effects on wild animal welfare. Wildlife encounters, such as whale- watching, seal-spotting, bird-watching, or tiger-tracking, may involve elements of disturbance or improper feeding of the target animals. The paper by Nunny and Simmonds brings up the need for strengthened legislation and guidelines to protect wild-living solitary sociable dolphins in relation to interactions with people.

In the area of wildlife conservation projects, a large range of activities from habitat restoration and head-starting programmes to translocation, captive breeding and the keeping of so-called “parallel populations” can be identified. When the focus is on species conservation, the welfare of the individual animals has historically often been given a lower priority. This has, however, changed during recent years, and scientists and others have raised questions about ethical aspects of such interventions and the potential to improve the welfare of animals involved in such projects (4–6). This aspect is highlighted in the paper by Beausoleil et al., which describes how cross-disciplinary information-sharing and collaborative research and practice in conservation can be applied in captive breeding projects, to facilitate the incorporation of both “fitness” and “feelings” to improve understanding of the welfare state of the animals.

Is there a difference between wildlife research and wildlife management regarding welfare aspects? In many countries, the legislative requirements differ depending on if the interventions are classified as research rather than management, although the actual handling of the animals may be identical. Lindsjö et al. argue for a more developed legislation about welfare matters in relation to these aspects.

Whilst aiming to improve conservation and indirectly improve the welfare status of animals, wildlife research, as well as captive breeding programmes for restoration of wild animal populations, can involve animal welfare risks (Figure 1). An increased interest in animal welfare can relate to various aspects of capture methods, the design of enclosures for breeding animals or head-started animals, preparation of captive-bred animals for a life in the wild, preparation of release-sites to improve the survival chances of newly-released animals, and proper post-release monitoring. In their paper, Greggor et al. highlight several of these aspects, emphasizing the need for an evidence-based approach to evaluate practices in conservation breeding facilities from an animal welfare perspective, while still meeting conservation goals. Thulin and Röcklinsberg analyse ethical considerations for wildlife reintroductions and rewilding projects, and Robins et al. discuss how telemetry can be used to improve post-release monitoring of apes. Arnemo et al. discusses long-term safety in bears equipped with radio transmitters, and Robins et al. do so in relation to orang-utans. The paper by de Jong addresses how, in accordance with the 3R principles, to avoid redundant handling and interventions. The 3R principles are commonly used when designing studies involving traditional laboratory animals for research. In wildlife research, this approach is yet to be further developed. Huber et al. focus on the possibility of using leukocyte coping capacity to quantify and evaluate stress in wildlife in captivity or when otherwise being handled by humans, and the strengths and weaknesses of this immunological approach.

**Figure 1:**
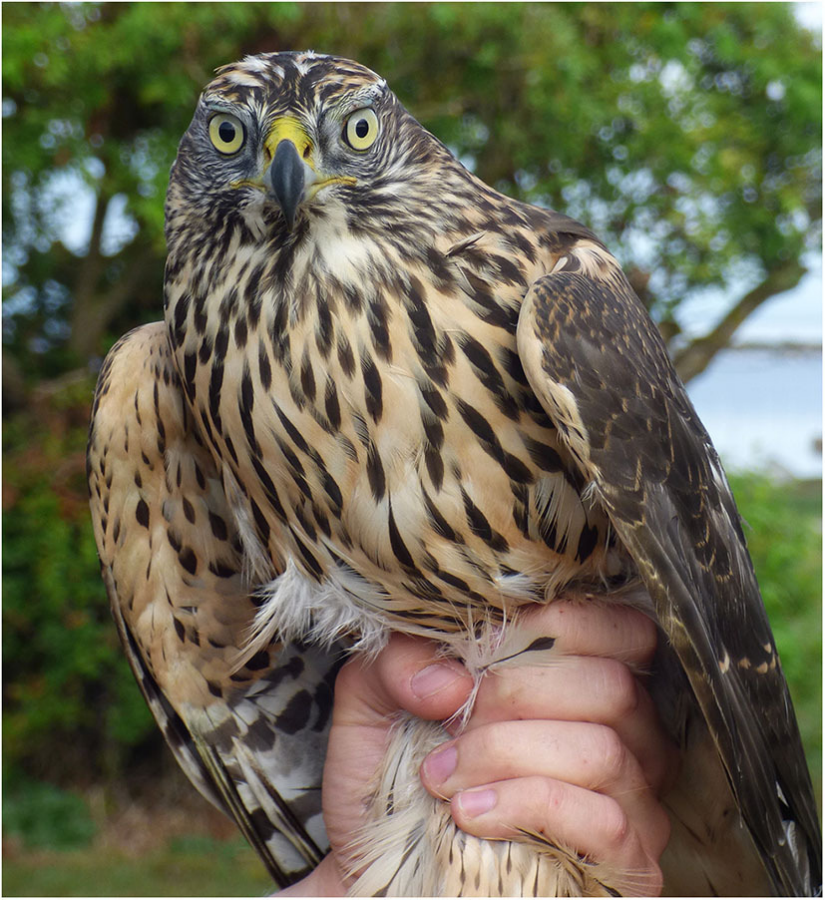
Young goshawk at ringing. A short moment of close interaction between humans and the bird. Such interactions can still, if not properly carried out, involve animal welfare risks.

The ultimate aim of captive breeding programmes is often to ensure that self-sustaining, free-ranging wildlife populations can exist, and this requires suitable habitat, sufficiently large enough areas, with intact ecosystems and sustainable ecosystem services. Furthermore, the choice of breeding animals in terms of health, behavior and temperament can be highly relevant for the welfare of their offspring, once released. Should suitable groups be formed before release? This aspect of sociality, and the importance of social networks for wildlife living in groups is discussed in the paper by Brakes. In addition to the welfare impacts for the translocated animals, the welfare of animals of other species at the release site should be considered: is there competition for food or other resources? Is a novel predator being released in an area?

If a wild animal is kept in captivity, for breeding, for education or show at a zoological garden, handled in research or for management purposes, there are both legal and moral obligations related to human responsibility for the well-being of the individual animal. This special issue highlights that this responsibility extends beyond the fence.

## Author Contributions

CB and HL have written the draft of the editorial and it was amended and revised by the other authors. All authors have served as editors of the Research Topic.

## Conflict of Interest

The authors declare that the research was conducted in the absence of any commercial or financial relationships that could be construed as a potential conflict of interest.
